# Flutamide ameliorates uterine decidualization and angiogenesis in the mouse hyperandrogenemia model during mid-pregnancy

**DOI:** 10.1371/journal.pone.0217095

**Published:** 2019-05-31

**Authors:** Han Gong, Weiqi Wu, Jingjie Xu, Dainan Yu, Bo Qiao, Hui Liu, Bei Yang, Yuezhen Li, Yan Ling, Haibin Kuang

**Affiliations:** 1 Department of Physiology, Basic Medical College, Nanchang University, Nanchang, Jiangxi, China; 2 Department of Obstetrics and Gynecology, West China Hospital, Sichuan University, Chengdu, Sichuan, China; 3 Jiangxi Provincial Key Laboratory of Reproductive Physiology and Pathology, Medical Experimental Teaching Center, Nanchang University, Nanchang, Jiangxi, China; 4 Department of Obstetrics and Gynecology, Jiangxi Province People’s Hospital, Nanchang, Jiangxi, China; South China Agricultural University, CHINA

## Abstract

**Background/Aim:**

Patients with polycystic ovary syndrome (PCOS), characterized by anovulation, hyperandrogenemia and polycystic ovaries, are still vulnerable to undergo recurrent pregnancy loss and premature labor even though the ovulatory process is pharmacologically recovered. However, its potential mechanism remains unknown. Thus, our aim was to investigate the effect and mechanism of hyperandrogenemia and flutamide (a non-steroidal anti-androgen) on the embryo implantation and pregnancy during mid-pregnancy.

**Methods:**

We used a mouse model in which PCOS-like hyperandrogenemia was induced by subcutaneous injection of testosterone propionate. In this model, we observed the effect of hyperandrogenemia and flutamide on the decidualization, angiogenesis and uNK cells by methods of immunohistochemistry, quantitative PCR, western blotting and Dolichos biflorus agglutinin (DBA) lectin staining.

**Results:**

Testosterone and flutamide treatment did not significantly influence the numbers of implanted embryo compared with the control group. However, different doses of testosterone significantly increased the ratio of resorbed /implanted embryo, decreased the level of *prl8a2* mRNA and cyclin D3 protein, inhibited the uterine angiogenesis and reduced the numbers of uNK cells, but combined treatment with flutamide markedly decreased the resorbed embryos, increased expressions of *prl8a2* mRNA and cyclin D3 protein and angiogenesis and numbers of uNK cells.

**Conclusion:**

Flutamide treatment can efficiently ameliorate the hyperandrogenemia-induced the disorders in aspects of decidualization, angiogenesis and uNK cells, which further improve the poor endometrial receptivity in PCOS patients.

## Introduction

Polycystic ovary syndrome (PCOS) is a common endocrine disease encountered among 4%-18% of women in the reproductive age all over the world [1–3]. Patients with PCOS manifest a constellation of symptoms including anovulation, hyperandrogenemia, polycystic ovaries and insulin resistance [1, 3–5]. Although it is heightened that anovulation is the primary reason for infertility in PCOS patients, they still present the impairment of endometrial receptivity and are vulnerable to undergo recurrent pregnancy loss and premature labor even though ovulatory process is pharmacologically recovered [5–6]. However, its potential mechanism is not clear. Excessive androgen secretion, caused by intrinsic ovarian disorder and hypothalamic-pituitary-ovarian axis abnormalities, is deemed to play an important role in the pathogenesis of endometrial receptivity. Insulin resistance is also contributed to hyperandrogenemia as they can directly stimulate androgen secretion of the ovary and adrenal gland or inhibit biosynthesis of sex hormone binding protein in the liver to increase bioavailability of free testosterone [7–8]. To relieve symptoms of hyperandrogenemia, Endocrine Society treatment guidelines currently advocate dietary and exercise modification followed by oral contraceptive pills (OCP) as the frontline treatment. When OCP is unable to fulfill the clinical expectations, antiandrogenic medication such as flutamide is employed as add-on therapy [4, 9], but little is known about the molecular mechanism of flutamide on the embryo implantation and pregnancy.

Substantial evidence indicated patients with PCOS had a dysregulated expression of angiogenic factors in their endometrium, including vascular endothelial growth factor (VEGF), angiopoietins, platelet-derived growth factor (PGF), transforming growth factor-β (TGF-β), and some basic fibroblast growth factors [10]. Uterine natural killer (uNK) cells are transient, ultimately differentiated and the most abundant lymphocytes present in human endometrium during pregnancy [11]. Instead of cytotoxic activity against tumor cells or virus-infected cells, uNK cells are considered as a vital source of cytokines to regulate trophoblast invasion, angiogenesis and decidualization in the uterus [12]. Immunohistochemistry and ELISA analysis showed that uNK cells secret multiple angiogenic factors, such as VEGF-A, VEGF-C, PGF and TGF-β [13]. Therefore, the formation of new decidual blood vessels and remodeling of existing vessels mainly depend on the normally functional uNK cells. Accumulating researches revealed female sex hormone progesterone and estrogen regulate recruitment, proliferation, differentiation and function of uNK cells with the aid of direct action on their nuclear receptors or intermediary cells [14]. However, the effect of hyperandrogenemia and flutamide on the angiogenesis, decidualization and uNK cells are less investigated.

Thus, the aim of this study was to investigate the effect of testosterone and flutamide on the embryo implantation and pregnancy during mid-pregnancy, under the condition of hyperandrogenemia. We used a mouse model in which PCOS-like hyperandrogenemia can be induced by subcutaneous injection of testosterone propionate [15–16]. In addition, we also observed the effect of hyperandrogenemia and flutamide on the decidualization, angiogenesis and the distribution and numbers of uNK cells in the uterus.

## Materials and methods

### Materials

Biotinylated-Dolichos biflorus agglutinins (DBA) lectin, acetyl-D-galactosamine, flutamide and rabbit anti-CD31 antibody were purchased from Sigma-Aldrich Chemical Company (St. Louis, MO, USA). Testosterone propionate was obtained from Shanghai General Pharmaceutical Co., Ltd. (Shanghai, China). Rabbit anti-cyclin D3 was purchased from Cell Signaling Technology (MA, USA). Diaminobenzidine solution and streptoavidin-peroxidase were obtained from Zhongshan Biotechnology (Beijing, China). Phosphatase inhibitor cocktail and nitrocellulose blotting membrane were provided by Applygen Technologies (Beijing, China).

### Animals and treatment

**Fig 1:**
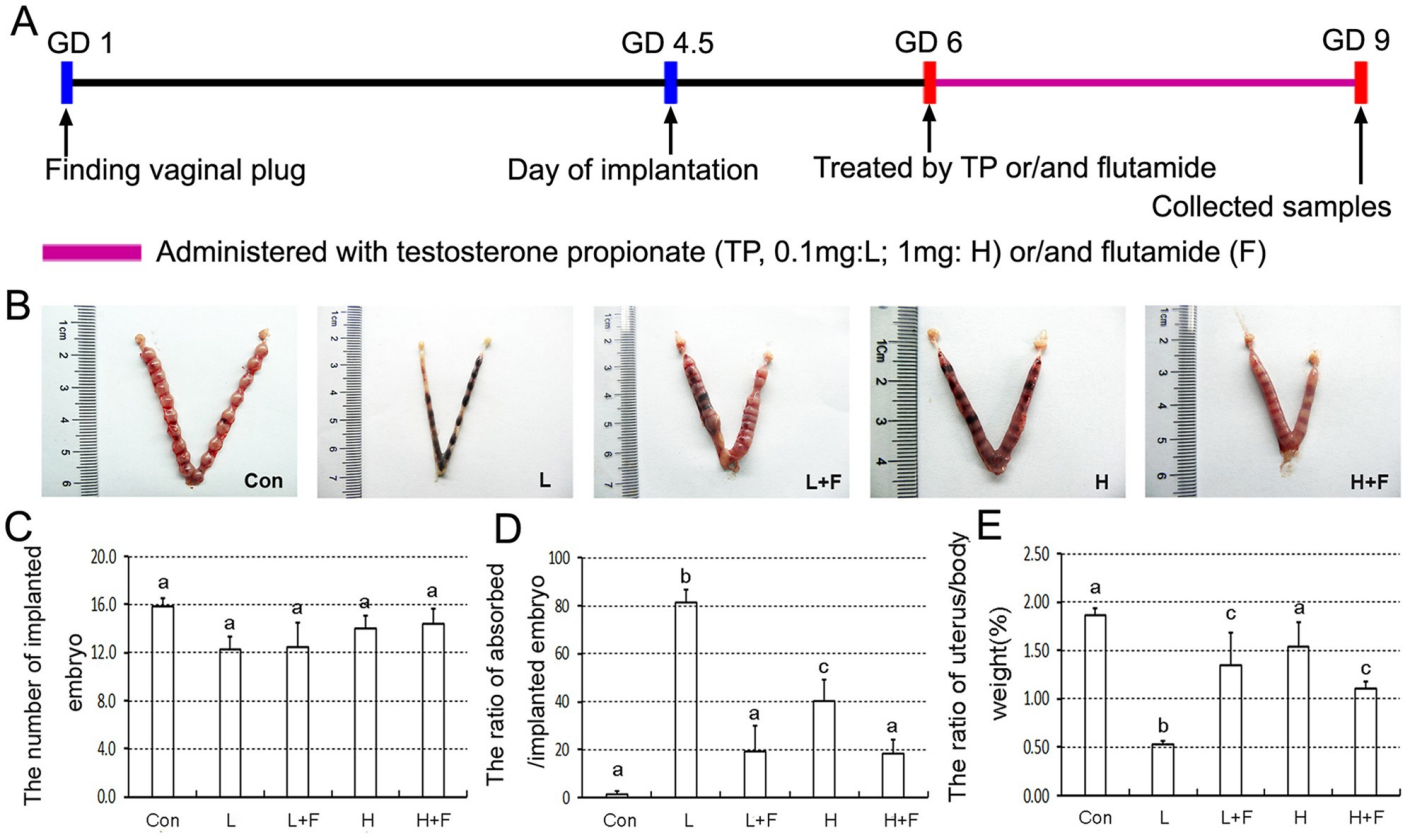
Effect of testosterone and flutamide on the embryo implantation and pregnancy. (A) Schematic representation of experimental design. Female and male mice were mated, and day of finding vaginal plug was regarded as gestational day (GD) 1. Pregnant mice were administered with testosterone propionate or/and flutamide from GD 6 to GD 9. Finally, the experimental mice were euthanized before cervical dislocation for samples collection between 14:00-15:00 on GD 9. (B) Representative pictures of implanted embryos in uteri. (C) The numbers of implanted embryo. (D) The ratio of resorbed embryo/implanted embryo. (E) The ratio of uterus/body weight; Results are shown as the mean ± SD of 10 animals. Groups with different superscript letters are statistically different (*P* < 0.05). Con, Control group; L, 0.1 mg testosterone propionate (TP)/per mouse; L+F, 0.1 mg TP + 0.15 mg flutamide /per mouse; H, 1 mg TP /per mouse; H+F, 1 mg TP + 0.15 mg flutamide /per mouse.

Mature female and male mice (Kunming strain, 9 weeks old, 25-30g) were obtained from the Animal Facility of Jiangxi traditional Medical University (Certificate number: JZDWNO2014-0054), and housed in a constant photoperiod (14/10 h light/dark cycle) and relative humidity (55±10%) at room temperature with food and water available ad libitum. All procedures of the experiment were approved by the Institutional Animal Care and Use Committee of Nanchang University (Permit Number: 20140293). The proposed sample size was calculated using the sample size formula with a power of 90% and an α-error of 0.05 [17]. The minimum number for each group was estimated to be n = 8. Additional animals were needed per group due to potential losses. Therefore, 10 mice per group were used. Virgin female mice were mated with fertile male mice of the same strain to achieve pregnancy (day of vaginal plug = gestation day 1, GD 1). The pregnant mice were randomly classified into the following 5 groups (n = 10/group): (1) Control group (Con, the same volume of sesame oil); (2) Low dose group (L): 0.1 mg testosterone propionate (TP) /per mouse; (3) 0.1 mg TP + 0.15mg flutamide/per mouse (L+F); (4) high dose group (H): 1 mg TP/per mouse; (5) 1 mg TP + 0.15mg flutamide/per mouse (H+F). The mice were injected subcutaneously in the back of neck with only TP or TP 30 min after flutamide administration once a day from GD 6 to GD 9 [15–16]. Treated mice were anesthetized with pentobarbital sodium before cervical dislocation for uterine collection between 14:00-15:00 on GD 9 (Fig 1A). Uteri of all mice described above were pictured by using a digital camera (Nikon, Japan) after dissection and isolation, and then were transferred into liquid nitrogen for storage and further analysis.

### RNA isolation and quantitative PCR

Total RNA was extracted from uterine tissues with RNAiso Plus solution (TaKaRa, China). Then total RNA samples were reverse-transcribed into single-stranded cDNA in a 25 μL reaction mixture (TaKaRa, China). Quantitative PCR was performed using a SYBR Premix Ex TaqIIMix according to manufacturer’s protocol (Takara, China). The results were terminally analyzed through ABI Prism 7500 software (Applied Biosystems, USA) and housekeeping 18s mRNA level was regarded as an internal control. The primers were as follows: 18s, 5’-AATCAGGGTTCGATTCCGGA-3’ (sense) and 5’-CCAAGATCCAACTACGAG CT-3’ (antisense); prl8a2, 5’- TTATGGGTGCATGGATCACTC-3’ (sense) and 5’-CCCACGTAAGGTCATCATGGA-3’ (antisense). Melting curve analysis and agarose gel electrophoresis were performed after the quantitative PCR assays to supervise the purity of PCR products.

### Immunohistochemistry

Isolated uterine tissues were cut into small blocks, fixed in Bouin’s solution for 24 h, dehydrated in ethanol, and embedded in liquid paraffin. Sections (5 μm) were cut, deparaffinized and rehydrated in ethanol solutions with a decreased concentration gradient. Then the slides were incubated in 3% hydrogen peroxide in PBS for 10min to inactivate endogenous peroxidase activity. Nonspecific binding was blocked in 5% bovine serum albumin (BSA) in PBS for 1h at room temperature. Next, the sections were added to rabbit anti-CD31 overnight at 4°C. After washing in PBS, the sections were incubated with secondary antibodies for 60 min at 37°C. The primary antibody was detected by fresh diaminobenzidine solution. Finally, sections were counterstained with Harris’ hematoxylin.

### uNK cells staining using DBA lectin histochemistry

uNK cells detection was performed as described previously [18]. Firstly, sections were deparaffinized in xylene, and rehydrated then treated with 3% hydrogen peroxide in PBS for 10 min to block endogenous peroxidase activity. Nonspecific binding was blocked in 1% BSA in PBS for 60min followed by an overnight incubation with biotinylated-DBA lectin at 4°C. The PBS washed sections were incubated with streptoavidin-peroxidase for another 50min at room temperature. After washing with PBS, The slides were incubated with fresh diaminobenzidine solution and counter-stained with Harris’ hematoxylin. Control sections were stained as above with the addition of 0.1 M N-acetyl-D-galactosamine to the DBA lectin incubation.

### Western blotting analysis

Uterine tissues were collected and lysed in the Tissue Protein Extraction Reagent supplemented with a phosphatase inhibitor cocktail and PMSF. The proper volume protein extract was subjected on the 12% SDS-polyacrylamide gel for electrophoresis, and then transferred onto the nitrocellulose blotting membrane. The membranes were blocked with 5% BSA for 1h at room temperature and incubated with rabbit anti-cyclin D3 primary antibodies (1:500, Cell Signaling, USA) at 4°C overnight. Membranes were washed in TBS containing 0.05% Tween-20 (TBST). The membranes were then incubated with horseradish peroxidase-conjugated secondary antibodies and visualized via enhanced chemiluminescence (Pierce, USA). Finally, the relative band intensity was measured by using the Quantity One software. The data were corrected for background, and normalized to GAPDH.

### Statistical analysis

The data are presented as the means ± SD. All statistical analyses were performed using Statistical Package for the Social Science (SPSS, Chicago, IL) 13.0 software.

Normal distribution of data was checked by Shapiro-Wilk test. The results were analyzed by one-way ANOVA followed by LSD’s post-hoc test. A value of *P* <0.05 was considered to be statistically significant.

## Results

### Effect of testosterone and flutamide on the embryo implantation and pregnancy

As shown in Fig 1B and 1C, Different doses of testosterone and their combined treatment with flutamide did not significantly influence the numbers of implanted embryo compared with the control group on GD 9. However, low and high-dose testosterone treatment group significantly increased the ratio of resorbed /implanted embryo, but combined treatment with flutamide markedly decreased the promoted effect of hypertestoterone on the ratio of resorbed /implanted embryo (Fig 1B and 1D).

### Effect of testosterone and flutamide on the uterine decidualization

**Fig 2:**
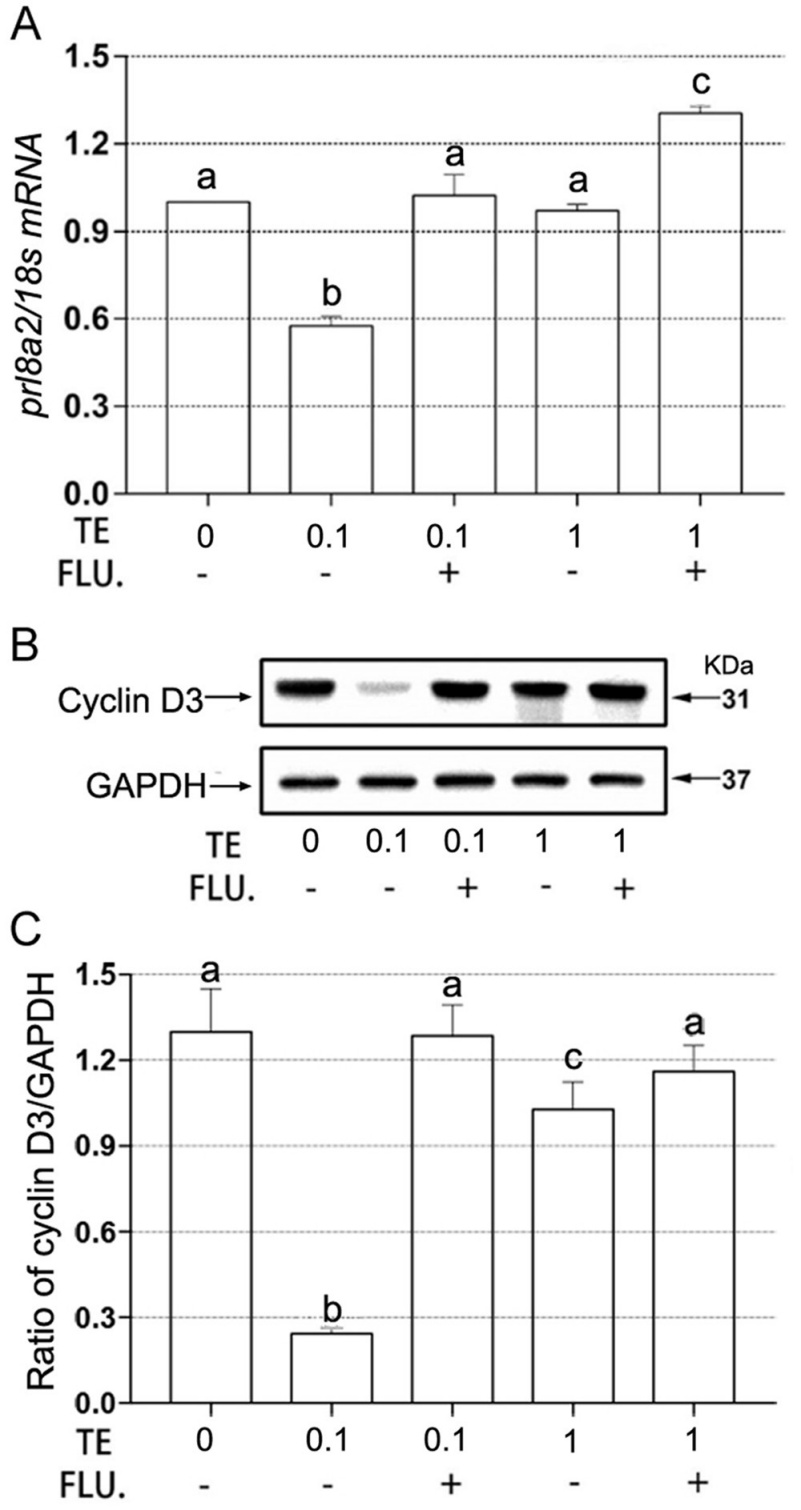
Effect of testosterone and flutamide on the uterine decidualization. (A) Expression levels of *prl8a2* mRNA in the uteri. (B) Representative western blotting result of cyclin D3 protein. (C) Densitometric values from western blotting analyses of cyclin D3 protein. mRNA level was quantified using quantitative PCR and normalized to 18s. Cyclin D3 protein was normalized to GAPDH expression. Results were shown as mean ±SD (n = 6 mice/treatment group). Groups with different superscript letters are statistically different (*P* < 0.05). TE, 0.1 mg or 1 mg testosterone propionate /per mouse; FLU, 0.15 mg flutamide /per mouse.

Compared to control group, combined treatment of testosterone and flutamide, and especially only low-dose testosterone treatment significantly reduced the ratio of uterus /body weight, yet high-dose testosterone had no significant effect on relative weights of uterus (Fig 1E). Furthermore, we further observed the mRNA or protein levels changes of decidualization markers, and quantitative PCR and western blotting results showed that a significant decrease in level of *prl8a2* mRNA and cyclin D3 protein was observed in low-dose testosterone treatment group compared with control group, but addition of flutamide can truly reverse this situation (Fig 2A–2C and S1 Fig). In addition, high-dose testosterone significantly reduced level of cyclin D3 protein, not *prl8a2* mRNA. However, level of cyclin D3 protein was returned to the control level after simultaneous flutamide treatment (Fig 2B and 2C, S1 Fig).

### Effect of testosterone and flutamide on the uterine angiogenesis and uNK cells

**Fig 3:**
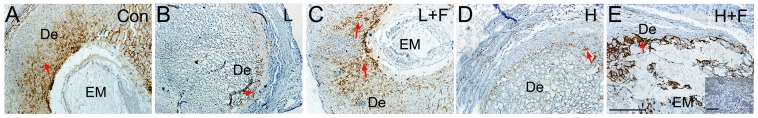
Effect of testosterone and flutamide on the uterine angiogenesis. Immunostaining of CD31 indicated the angiogenesis of uteri in mice. Brown-yellow staining indicates positive signals (red arrows). (A) Con, Control group; (B) L, 0.1 mg testosterone propionate (TP) /per mouse; (C) L+F, 0.1 mg TP + 0.15 mg flutamide/per mouse; (D) H, 1 mg TP/per mouse; (E) H+F, 1 mg TP + 0.15 mg flutamide/per mouse. Inset of E is negative control (primary antibodies was replaced by normal rabbit serum IgG). EM: embryo; De: deciduas. Scale bars, 50μm.

CD 31 immunostaining indicated treatment with different doses of testosterone significantly inhibited the angiogenesis of uteri in mice relative to control group (Fig 3A, 3B and 3D). However, simultaneous administration of flutamide markedly increased the CD 31 staining and displayed the same normal uterine angiogenesis as the control mice (Fig 3A, 3C and 3E).

**Fig 4:**
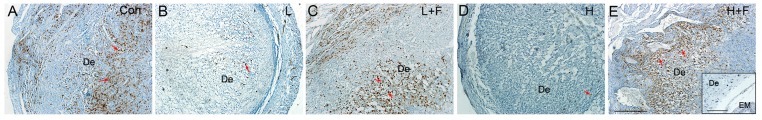
Effect of testosterone and flutamide on the uNK cells. Immunostaining of DBA lectin indicated the uNK cells of uteri in mice. Brown-yellow staining indicates positive signals (red arrows). (A) Con, Control group; (B) L, 0.1 mg testosterone propionate (TP) /per mouse; (C) L+F, 0.1 mg TP + 0.15 mg flutamide/per mouse; (D) H, 1 mg TP/per mouse; (E) H+F, 1 mg TP + 0.15 mg flutamide/per mouse. Inset of E is negative control (the addition of N-acetyl-D-galactosamine to the DBA lectin incubation). EM: embryo; De: deciduas. Scale bars, 50μm.

As shown in Fig 4A, DAB lectin staining indicated that the uNK cells were increasingly accumulated and distributed in large quantities at the mesometrial side of normal implantation sites. Nevertheless, the positive signals of DAB lectin staining were significantly down-regulated after testosterone treatment especially high-dose group compared with control group (Fig 4B and 4D). Nevertheless, combined treatment with flutamide significantly increased the numbers of uNK cells and recovered the normal distribution of uNK cells in the uteri of pregnant mice (Fig 4C and 4E).

## Discussion

Numerous studies have illustrated that infertile PCOS patients present the impairment of endometrial receptivity and were vulnerable to undergo recurrent pregnancy loss, premature labour and fetal growth retardation [2, 6]. However, exact mechanism involving it still needed further investigation. uNK cells play an important role in secreting cytokines to regulate the angiogenesis and decidualization at maternal-fetal interface during pregnancy [14]. To the best of our knowledge, our experiment was the first to confirm that the impairment of endometrial receptivity included in PCOS patients was originated in poor angiogenesis, which is caused by hyperandrogenemia-induced decrease in uNK cells. The results showed that hyperandrogenemia significantly increased the ratio of resorbed embryos on GD 9, with concomitant down-regulation in the numbers of uNK cells and blood vessels in the endometrium in a dose-dependent manner. Gradual disorder in construction of endometrial blood vessels was also found as dose of external testosterone increased on GD 9. In addition, external testosterone administration inhibited the expression of cyclin D3 protein and *prl8a2* mRNA. Nevertheless, antiandrogenic drug flutamide could efficiently ameliorate all the dysregulation mentioned above back to normal level.

It was reported that decidual neoangiogenesis around the embryonic crypt started at GD 5 in mice, shortly 12 h after implantation [13]. Around GD 9-13, fetal capillaries branching from umbilical vessels bathed into maternal blood supply to form mature hemochorial placenta and utero-placental circulation was opened to supply blood and nutrients to fetus [19]. Our experiment found external testosterone treatment significantly reduce immunohistostaining signal of CD31 in a dose-dependent manner on GD 9, especially in high-dose testosterone treatment group, completely circular blood vessels were barely observed. This is almost consistent with Zhao *et al*.’s result that the mRNA and protein expression level of VEGF were significantly inhibited in PCOS patient group compared with control group [20]. uNK cells are most prominent population of lymphocytes present in pregnant deciduas during early pregnancy, histological studies reveal that they are spatially and temporally distributed within the area of newly developing microvasculature and secret multiple angiogenic factors to direct aspects of formation of supportive vascular networks [21]. Gibson *et al*. showed a critical role of estrogen in regulating secretion of CCL2 to mediate endometrial angiogenesis [22]. Furthermore, Kim *et al*. illustrated that progesterone could be essential for VEGFA production in DBA^+^ uNK cells [23]. However, our study firstly demonstrated that testosterone damaged endometrial angiogenesis in a dose-dependent manner, which might be explained by the decrease in the number of uNK cells. Since the researches related to the regulation of testosterone on the uNK cells are limited, the mechanism underlying it awaits characterization.

We also investigated whether poor endometrial receptivity present in PCOS patients is correlated with the decidualization of endometrial stromal cells during pregnancy. Pregnant mice supplied with external testosterone actually resulted in a dysregulated expression of decidualization markers, such as depressed expression of cyclin D3 protein and *prl8a2* mRNA on GD 9. It is noted that cyclin D3, as a G1 phase cell cycle regulator included in stromal cell proliferation and decidualization, is also significantly decreased in Hoxa-10 knockout mice [24]. Hoxa-10 is a homeobox transcription factor essential for developmental control of endometrial differentiation and receptivity [25]. Cermik *et al*. found testosterone treatment could reduce expression of Hoxa-10 in a human endometrial adenocarcinoma cell line and the authors also observed the negative impact in endometrium of PCOS patients with hyperandrogenemia [26]. In addition, Sroga *et al*.’s experiment showed adenovirus-driven cyclin D3 replacement in Hoxa-10 knockout mice improved stromal cell decidualization and prolonged pregnancy until day 10 of pregnancy [24]. However, continued adenoviral delivery of cyclin D3 to support normal decidualization in defect endometrium was flawed, because repeated administration can result in animal sickness and even lethal to pups before delivery [24]. Other common pharmacological options for hyperandrogenemia present in PCOS patients are involved in dexamethasone, metformin and flutamide [2]. Through incubating endometrial cells of normal cycling women, dexamethasone was verified to restore the impairment of a low-dose testosterone on endometrium, but had no effect on high dose. Furthermore, metformin treatment impaired expression of prolactin in the endometrial cells in a dose-dependent manner and this phenomenon got worse in hyperandrogenaemic environment, which cannot be reversed by dexamethasone [27]. Nevertheless, our experiment pointed out another antiandrogenic drug flutamide can restore expression level of cyclin D3 protein and *prl8a2* mRNA in endometrium cells regardless of testosterone concentration and it provided a novel drug to treat defect decidualization present in PCOS patients.

In conclusion, our results provide evidence that hypertestosterone significantly increased the ratio of resorbed embryo, decreased uterine decidualization, and inhibited the uterine angiogenesis and reduced the numbers of uNK cells. However, simultaneous administration of flutamide can effectively ameliorate uterine decidualization and angiogenesis via up-regulation of prl8a2, cyclin D3, CD 31 expression and numbers of uNK cells in the mouse uteri during mid-pregnancy. Future work will be concentrated on elucidating the exact mechanism underlying these hyperandrogenemia induced dysregulation and possible combined therapy with flutamide on treating PCOS patients.

## Supporting information

S1 Fig Effect of testosterone and flutamide on level of uterine cyclin D3 protein. Uncropped and unaltered western blots results of cyclin D3 protein (31 KDa) and GAPDH (37 KDa).
(TIF)
