# Supplementary figures and images for: Flutamide ameliorates uterine decidualization and angiogenesis in the mouse hyperandrogenemia model during mid-pregnancy
Source: PLoS One. 2019 May 31;14(5):e0217095. doi: 10.1371/journal.pone.0217095 (PMC6544220; doi:10.1371/journal.pone.0217095)

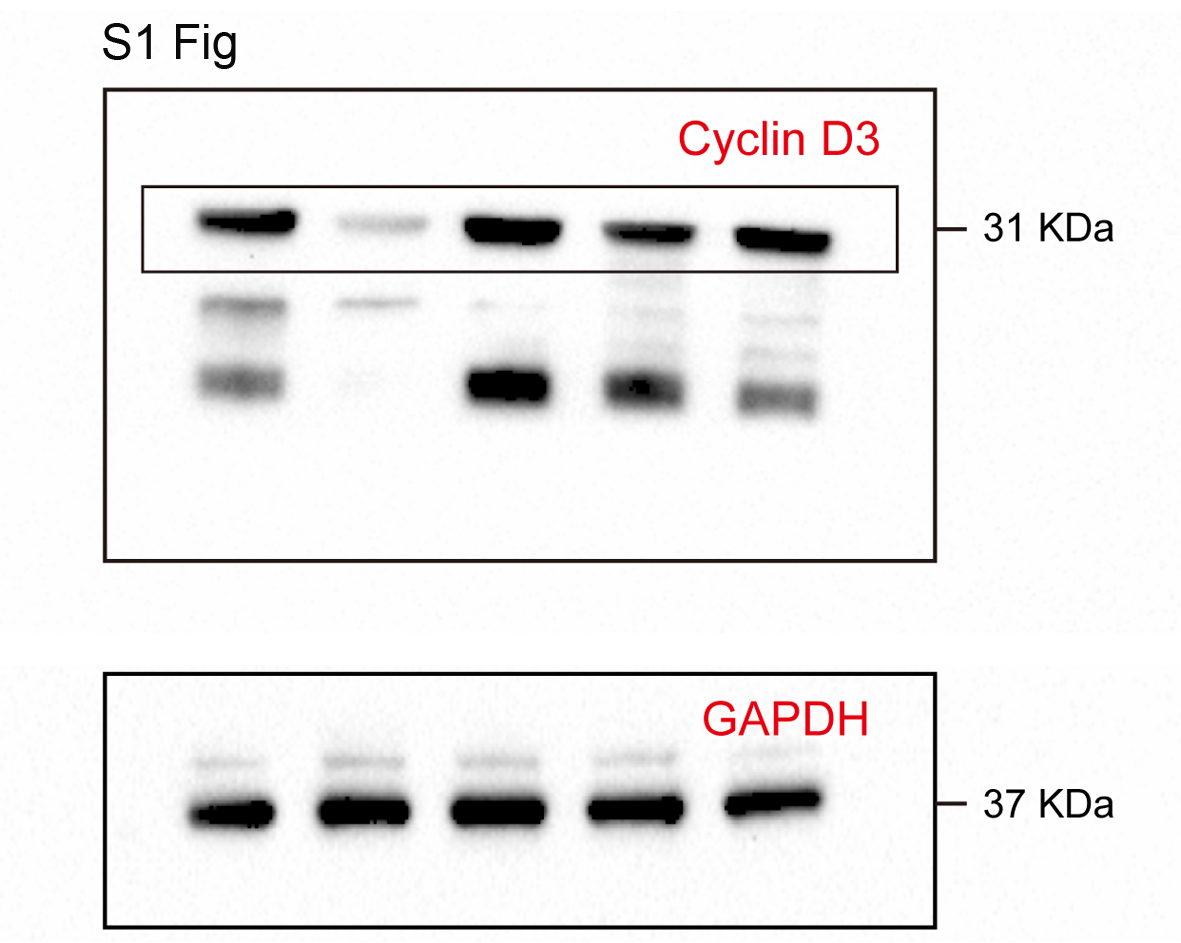

Supplement: S1 Fig — Uncropped and unaltered western blots results of cyclin D3 protein (31 KDa) and GAPDH (37 KDa). (TIF) [file pone.0217095.s001.tif]
